# Changes in implant choice and surgical technique for hemiarthroplasty: 21,346 procedures from the Swedish Hip Arthroplasty Register 2005–2009

**DOI:** 10.3109/17453674.2011.641104

**Published:** 2012-02-08

**Authors:** Olof Leonardsson, Göran Garellick, Johan Kärrholm, Kristina Åkesson, Cecilia Rogmark

**Affiliations:** ^1^Department of Ortopaedics, Lund University, Skåne University Hospital, Malmö; ^2^the Swedish Hip Arthroplasty Register, Registercentrum VGR, Göteborg, Sweden

## Abstract

**Background and purpose:**

Treatment of displaced femoral neck fractures in Sweden has shifted towards more arthroplasties, especially hemiarthroplasties. We describe the hemiarthroplasty population in Sweden 2005 through 2009.

**Methods:**

Since 2005, the Swedish Hip Arthroplasty Register has registered hemiarthroplasties on a national basis. We assessed hemiarthroplasty procedures in the Register 2005–2009 regarding patient details, implants, and surgical techniques. Completeness of recordings was calculated compared to the Swedish National Patient Register.

**Results:**

Completeness increased from 89% to 96% during the study period. 21,346 hemiarthroplasty procedures were assessed. The relative number of patients with femoral neck fracture as diagnosis increased from 91% to 94%; the proportion of men increased from 27% to 30%. The median age increased from 83 to 84 years in men and from 84 to 85 years in women. Patients classified as having evident cognitive impairment increased from 19% to 22%. More men than women were ASA 4. The proportion of monoblock-type implants (Austin-Moore and Thompson) decreased from 18% to 0.9%. Modular implants increased generally, but in 2009 bipolar implants decreased in favor of unipolar implants. Lubinus and Exeter stems, and Mega Caput and Vario Cup implant heads were most common. The use of uncemented implants decreased from 10% to 3%. Use of the anterolateral approach increased from 47% to 56%.

**Interpretation:**

Important changes in surgical technique and implant choice occurred during the observation period. We interpret these changes as being reflections of the continuing effort by Swedish orthopedic surgeons to improve the quality of treatment, because the changes are consistent with recent findings in the Swedish Hip Arthroplasty Register and in other scientific studies.

In Sweden, approximately 6,500 patients annually sustain displaced femoral neck fractures (Swedish National Board of Health and Welfare 2010, Thorngren 2010). Scandinavia has had a strong tradition in treating these fractures with closed reduction and internal fixation. During the past decade, there has been a shift towards more arthroplasties (Rogmark et al. 2010). In 2009, approximately 3 out of 4 displaced femoral neck fractures in Sweden were treated with arthroplasties and the vast majority of them where hemiarthroplasties (Thorngren 2010).

Founded in 1979, the Swedish Hip Arthroplasty Register (SHAR) includes total hip arthroplasties (THAs) performed because of osteoarthritis, rheumatoid arthritis, and fracture of the femoral neck (Garellick et al. 2010). To supplement the information on fracture treatment, hemiarthroplasty registration was established as a part of the SHAR in 2005. Globally, there are a number of registries dedicated to total hip arthroplasty. In addition to the SHAR, official national recording of hemiarthroplasties with an acceptable degree of completeness and annual reports only happens in Norway (Furnes and Gjertsen 2010) and Australia (Graves et al. 2010).

Important goals of the Swedish Hip Arthroplasty Register are to analyze hip arthroplasty surgery and to identify factors that predict the outcome. Here we describe the hemiarthroplasty population in Sweden and the changes that occurred during the first 5 years of the register.

## Patients and methods

The Swedish Hip Arthroplasty Register (SHAR) has 100% coverage (participating hospitals) and 98% completeness (registration on an individual basis) regarding THAs (Garellick et al. 2010). Since January 1, 2005, hemiarthroplasties have been registered as part of the SHAR. The data are collected by a contact secretary at each unit after surgery and reported to the SHAR through the internet. In accordance with the Swedish Data Act, all patients receive information and are free to renounce their participation in the registration at any point.

All records in the SHAR are linked to the patients by the individual and unique 10-digit identity number given to all Swedish citizens at birth and to immigrants after entering the country. The registration includes hemiarthroplasties regardless of diagnosis. Primary procedures for femoral neck fractures and salvage procedures after internal fixation are most common. Surgical details (i.e. surgical approach, and type of implant and fixation) and clinical data (i.e. age, sex, and—most recently—American Society of Anesthesiologists (ASA) grade) are recorded. The presence of cognitive impairment is classified by the surgeon in a simple manner as “none”, “suspected”, or “evident”. The SHAR also registers re-operations including open reductions of dislocations. For any re-operation, the medical records are examined by the SHAR coordinating secretaries and approximately 100 additional variables are introduced into the registration.

In addition to publishing annual reports, the SHAR holds annual seminars (both in cooperation with the Swedish Orthopaedic Association and in its own right) to inform the orthopedic community of new findings and results, and to influence the choice of implants and surgical techniques.

The degree of coverage and the degree of completeness of hemiarthroplasty registration were determined for 2005, 2006, 2008, and 2009 by comparison with the Swedish National Patient Register (NPR; National Board of Health and Welfare). The NPR records the diagnoses and surgical procedures (ICD-10, Nordic Surgical Procedure Code) of all in-patient episodes and also all out-patient visits in Sweden, and links them to the patient's identity number. It is a legal obligation to report to the NPR (Swedish National Board of Health and Welfare 2010). Comparing the SHAR database and the NPR database at the level of the individual (through the patient's identity number), each of the entries in the two databases fell into one of the following categories: (1) a matched individual, i.e. a patient who was registered in both registers, (2) an individual who was only registered in the SHAR, and (3) an individual who was only registered in the NPR. The degree of completeness of the SHAR was calculated from numbers of patients in the 3 different categories: 1 + 2 divided by 1 + 2 + 3.

### Statistics

We used SPSS software versions 15.0 and 17.0 for the statistical calculations. In order to give an accurate view on the changes in patient ages during the study period, we report the median age with range for men and women for each year.

## Results

### Coverage and completeness

The calculated coverage (participating units) was 100% throughout the period (analyzed in 2005, 2006, 2008 and 2009). The completeness (hemiarthroplasty procedures) was 89% in 2005 but had increased to 96% in 2006. This level of completeness was then maintained throughout the period 2008–2009 (Table 1).

**Table 1.** Baseline characteristics

	2005	2006	2007	2008	2009	Whole period
	n (%)	n (%)	n (%)	n (%)	n (%)	n (%)
Sex						
Female	2,824 (73)	3,123 (74)	3,064 (72)	3,166 (71)	3,126 (70)	15,303 (72)
Male	1,045 (27)	1,119 (26)	1,202 (28)	1,321 (29)	1,356 (30)	6,043 (28)
Total	3,869	4,242	4,266	4,487	4,482	21,346
Completeness of procedure						
recordings, %	89	96		96	96	
Mean age, years	83	83	84	84	84	84
Side						
Left	2,070 (54)	2,191 (52)	2,231 (52)	2,348 (52)	2,314 (52)	11,154 (52)
Right	1,799 (46)	2,051 (48)	2,035 (48)	2,139 (48)	2,168 (48)	10,192 (48)
Diagnosis						
Acute FNF **^a^**	3,533 (91)	3,938 (93)	4,005 (94)	4,230 (94)	4,225 (94)	19,931 (93)
Failed internal fixation	263 (7)	226 (5)	183 (4)	172 (4)	165 (4)	1,009 (5)
Other **^b^**	73 (2)	78 (2)	78 (2)	85 (2)	92 (2)	406 (2)
Hospital type						
University/Regional	957 (25)	1,262 (30)	1,220 (29)	1,219 (27)	1,255 (28)	5,913 (28)
Central	2,242 (58)	2,357 (56)	2,425 (57)	2,576 (57)	2,508 (56)	12,108 (57)
Rural	669 (17)	623 (15)	621 (15)	692 (15)	719 (16)	3,324 (16)
Private	1 (0)					1 (0)

**^a^** Acute femoral neck fracture(including 298 other fractures around the hip)

**^b^** Including neoplasms, idiopathic avascular necrosis, and osteoarthritis.

### Procedure rates

For the whole period 2005–2009, 21,346 hemiarthroplasty procedures were reported. During this period, the number of hemiarthroplasties reported to the SHAR increased by 16%—to 4,482 procedures in 2009 (Table 1). Adjusting for the difference in completeness over time, the increase in hemiarthroplasty procedures during the study period was 7%.

Hemiarthroplasty surgery was performed in 65 hospitals and 57 of the hospitals performed more than 10 procedures per year. The majority of procedures (57%) were performed at central hospitals, while university/regional hospitals and rural hospitals accounted for 28% and 16%, respectively (Table 1).

### Patients

From 2005, the proportion of men increased from 27% to 30% (Table 1). During the same period, the median age of men increased from 83 (39–98) to 84 (10–100) years and that of women increased from 84 (46–101) to 85 (44–102) years (Table 2). The proportion of patients over 85 years of age increased from 40 to 47 %.

**Table 2.** Median age, cognitive impairment, and ASA grade, by year and by sex

	Total no.	Median age (range)	Cognitive impairment **^a^**, n (%)			ASA grade **^b^**, n (%)				
			none	suspected	evident	I	II	III	IV	V
2005										
Female	2,824	84 (46–101)	1,609 (72)	178 (8)	435 (20)					
Male	1,045	83 (39–98)	593 (72)	79 (10)	148 (18)					
2006										
Female	3,123	84 (42–101)	1,767 (70)	253 (10)	513 (20)					
Male	1,119	84 (44–102)	664 (72)	101 (11)	156 (17)					
2007										
Female	3,064	85 (45–104)	1,737 (68)	286 (11)	545 (21)					
Male	1,202	84 (51–100)	679 (67)	141 (14)	201 (20)					
2008										
Female	3,166	85 (42–102)	1,907 (69)	250 (9)	595 (22)	65 (3)	1,087 (44)	1,193 (48)	128 (5)	3 (0.1)
Male	1,321	84 (21–102)	761 (68)	124 (11)	230 (21)	25 (2)	356 (35)	574 (56)	64 (6)	1 (0.1)
2009										
Female	3,126	85 (44–102)	1,830 (68)	257 (10)	605 (22)	88 (3)	1,213 (44)	1,340 (48)	138 (5)	2 (0.1)
Male	1,356	84 (10–100)	829 (69)	124 (10)	253 (21)	29 (2)	378 (32)	674 (57)	91 (8)	2 (0.2)
Whole period **^c^**										
Female	15,303	85 (42–104)	8,850 (69)	1,224 (10)	2,693 (21)	153 (3)	2,300 (44)	2,533 (48)	266 (5)	5 (0.1)
Male	6,043	84 (10–102)	3,526 (69)	569 (11)	988 (19)	54 (2)	734 (33)	1,248 (57)	155 (7)	3 (0.1)

**^a^**Grading of cognitive impairment missing for 3,496 patients during the whole period.

**^b^** ASA grading missing for 1,518 patients 2008–2009.

**^c^** For ASA grade, only records for 2008 and 2009 are included.

In 2009, acute femoral neck fractures accounted for 94% of the recordings, which was an increase since 2005 (91%). The proportion of procedures that were attributable to failed internal fixation decreased during the study period from 7% to 4% (Table 1).

Between 2005 and 2009, the presence of grading of cognitive impairment increased from 79% to 87% of patients. About 21% of the female patients with recordings of grade of cognitive impairment and 19% of the males were classified into the group with evident cognitive impairment (Table 2). In the whole group, patients classified with evident cognitive impairment increased from 19% in 2005 to 22% in 2009.

By 2008, ASA grading had become fairly well established and was reported in 78% of the cases. The following year, ASA completeness increased to 88%. Despite their lower age, more male patients than female patients (7% as compared to 5%) were classified as ASA grade 4 (severe systemic disease that is a constant threat to life) during 2008–9. Furthermore, during the same period 47% of the women were classified as ASA grade 1 or 2 (normal, healthy patients or patients with mild systemic disease) as compared to 36% of the men (Table 2).

### Implants and surgical technique

**Figure 1.**
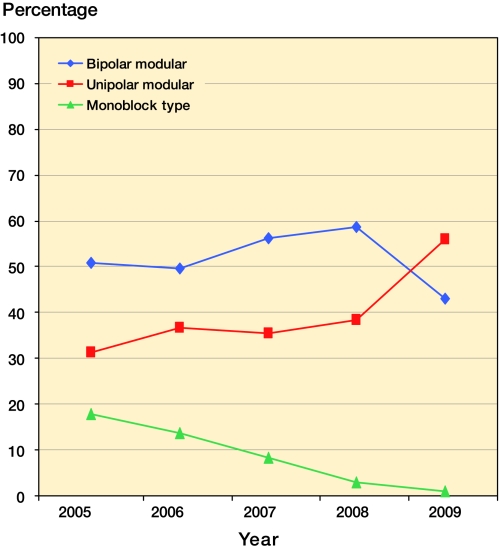
Proportion of implant types used over time.

Since 2005, there has been a gradual and consistent decrease in the use of monoblock-type implants (i.e. implants manufactured and delivered in one piece without the possibility of modification). In 2005, monoblock-type implants constituted 18% of the procedures and in 2006 the proportion was 14%. In 2009, this type of implant accounted for only 0.9% of the procedures. A general increase in the use of both unipolar and bipolar modular implants could be seen from 2005 through 2008. In 2009, however, the use of unipolar modular implants increased above the level of use of bipolar modular implants (56% vs. 43%) (Figure 1). The most commonly used unipolar and bipolar implant heads were the Mega Caput (in Sweden, recently renamed LINK Unipolar Head) (43%) and the Vario Cup (50%). Use of the Vario Cup decreased in 2009 compared to 2008 (41% vs. 53%) whereas use of the Mega Caput increased from 41% to 47% during the same period (Table 3).

**Table 3.** Most common implant heads

	2005	2006	2007	2008	2009	Whole period
Implant head	n (%)	n (%)	n (%)	n (%)	n (%)	n (%)
Unipolar **^a^**						
Mega Caput	463 (38)	655 (42)	681 (45)	705 (41)	1,174 (47)	3,678 (43)
V40 Unipolar	277 (23)	333 (21)	377 (25)	497 (29)	710 (28)	2,194 (26)
Unipolar Head	337 (28)	449 (29)	228 (15)	152 (9)	180 (7)	1,346 (16)
Various other designs	133 (11)	119 (8)	230 (15)	373 (22)	449 (18)	1,304 (15)
Unipolar total	1,210	1,556	1,516	1,727	2,513	8,522
Bipolar **^a^**						
Vario Cup	1,011 (51)	1,053 (50)	1,320 (55)	1,381 (53)	795 (41)	5,560 (50)
UHR Universal Head	604 (31)	583 (28)	638 (27)	709 (27)	674 (35)	3,208 (29)
Ultima Monk	316 (16)	435 (21)	388 (16)	429 (16)	325 (17)	1,893 (17)
Various other designs	37 (2)	36 (2)	50 (2)	111 (4)	132 (7)	366 (3)
Bipolar total	1,968	2,107	2,396	2,630	1,926	11,027
Monoblock type	690	577	354	129	40	1,790
Tumor implant		1				1
Missing	1	1		1	3	6
Total	3,869	4,242	4,266	4,487	4,482	21,346

**^a^** Mega Caput: Waldemar Link, Hamburg, Germany; V 40 Unipolar: Stryker, Kalamazoo, MI; Unipolar Head: Smith & Nephew Orthopaedics, Memphis, TN; Vario Cup: Waldemar Link; UHR Universal Head: Stryker; Ultima Monk: Zimmer, Warsaw, IN.

Over the entire observation period, the Lubinus SPII stem (43%) and the Exeter Polished stem (25%) were the most commonly used stems. The CPT (CoCr), the Spectron EF Primary, the Thompson, and the Austin-Moore stems each accounted for between 3% and 6%. However, the use of Thompson stems decreased from 9% in 2005 to 1% in 2009 and the use of Austin-Moore stems decreased from 9% to 0.6% (Table 4). Some of the Thompson and Austin-Moore stems were used as modular implants.

**Table 4.** Distribution of stem types

	2005	2006	2007	2008	2009	Whole period
Stem **^a^**	n (%)	n (%)	n (%)	n (%)	n (%)	n (%)
Lubinus SP II	1,456 (38)	1,665 (39)	1,966 (46)	2,095 (47)	1,957 (44)	9,148 (43)
Exeter Polished	870 (22)	936 (22)	1,040 (24)	1,204 (27)	1,377 (31)	5,427 (25)
CPT (CoCr)	187 (5)	211 (5)	240 (6)	275 (6)	336 (7)	1,249 (6)
Spectron EF Primary	351 (9)	408 (10)	182 (4)	107 (2)	163 (4)	1,211 (6)
Thompson	354 (9)	360 (8)	244 (6)	168 (4)	44 (1)	1,170 (5)
Austin-Moore	329 (9)	220 (5)	78 (2)	23 (0.5)	26 (0.6)	676 (3)
MS30 Polished	0 (0)	1 (0)	111 (3)	177 (4)	163 (4)	452 (2)
Corail	26 (0.7)	96 (2)	92 (2)	109 (2)	94 (2)	417 (2)
Covision straight	0 (0)	0 (0)	24 (0.6)	152 (3)	233 (5)	409 (2)
ETS Endo	98 (3)	104 (2)	129 (3)	48 (1)	0 (0)	379 (2)
Various other designs	198 (5)	241 (6)	160 (4)	129 (3)	89 (2)	808 (4)
Total	3,869	4,242	4,266	4,487	4,482	21,346

**^a^** Lubinus SP II: Waldemar Link, Hamburg, Germany, Exeter polished: Stryker, Kalamazoo, MI; CPT (CoCr): Zimmer, Warsaw, IN; Spectron EF Primary: Smith & NephewOrthopaedics, Memphis, TN; Thompson: Various manufacturers, e.g. Corin Ltd, Cirencester, UK; Austin-Moore: Various manufacturers, e.g. Stryker, Kalamazoo, MI; MS30 Polished: Zimmer, Winterthur, Switzerland; Corail: Depuy, Saint Priest, France; Covision straight: Covision Medical Technologies, Carlton in Lindrick, UK; ETS Endo: Stryker, Kalamazoo, MI.

The 2 dominating stem-head combinations in the unipolar group were Lubinus SP II with Mega Caput and Exeter Polished with V40 Unipolar (69%); in the bipolar group, the corresponding combinations were Lubinus SP II with Vario Cup and Exeter Polished with UHR Universal Head (77%). Consistent with the decrease in the use of the Vario Cup implant head in 2009, there was a decrease in the use of the combination Lubinus SP II stem with Vario Cup head in the same year (Table 5).

**Table 5.** The most common stem-head combinations

	2005	2006	2007	2008	2009	Whole period
Stem with head	n (%)	n (%)	n (%)	n (%)	n (%)	n (%)
Unipolar						
Lubinus SP II w. Mega Caput	463 (38)	655 (42)	680 (45)	704 (41)	1,162 (46)	3,664 (43)
Exeter Polished w. V40 Unipolar	276 (23)	327 (21)	375 (25)	497 (29)	709 (28)	2,184 (26)
Spectron EF Primary w. Unipolar Head	333 (28)	398 (26)	177 (12)	93 (5)	106 (4)	1,107 (13)
Various other combinations	138 (11)	176 (11)	284 (19)	433 (25)	536 (21)	1,567 (18)
Unipolar total	1,210	1,556	1,516	1,727	2,513	8,522
Bipolar						
Lubinus SP II w. Vario Cup	978 (50)	1,004 (48)	1,279 (53)	1,366 (52)	785 (41)	5,412 (49)
Exeter Polished w. UHR Universal Head	564 (29)	564 (27)	629 (26)	698 (27)	659 (34)	3,114 (28)
CPT (CoCr) w. Ultima Monk	186 (9)	206 (10)	170 (7)	169 (6)	211 (11)	942 (9)
Various other designs	240 (12)	333 (16)	318 (13)	397 (15)	271 (14)	1,559 (14)
Bipolar total	1,968	2,107	2,396	2,630	1,926	11,027
Total	3,869	4,242	4,266	4,487	4,482	21,346

**Figure 2.**
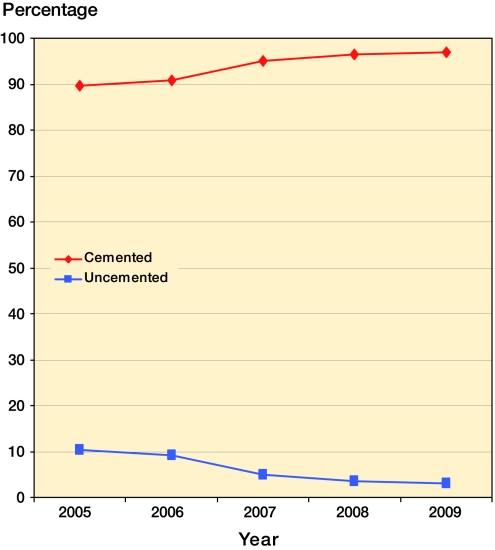
Proportion of methods of implant fixation used over time.

The total proportion of procedures performed with uncemented implants was 6%, but this choice of fixation decreased from 10% in 2005 to 3% in 2009 (Figure 2).

**Figure 3.**
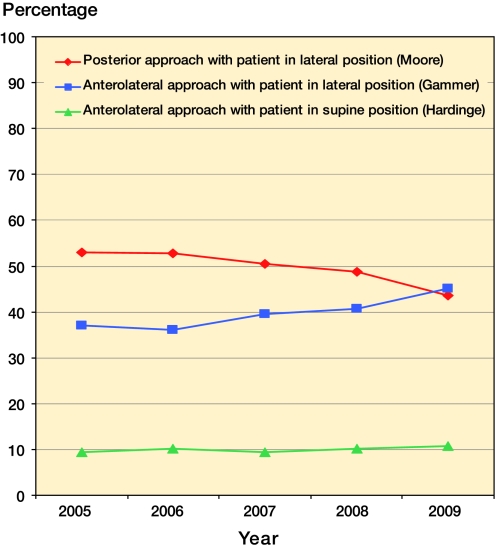
Proportion of surgical approaches used over time.

3 surgical approaches were used in 99% of all procedures: the posterior approach with the patient in a lateral position (Moore 1957), the anterolateral approach with the patient in a lateral position (Gammer 1985), and the anterolateral approach with the patient in a supine position (Hardinge 1982). Between 2005 and 2009, the number of procedures performed using an anterolateral approach (Gammer and Hardinge together) increased from 47% to 56% whereas the number using the posterior approach decreased from 53% to 44% (Figure 3).

## Discussion

We found important changes in implants and technique during the first 5 years of SHAR hemiarthroplasty registration. Most important was the rapid decrease in use of monoblock-type implants and prostheses implanted without cement, and also the gradually increasing use of the anterolateral surgical approach together with the decreasing use of bipolar implants in 2009.

In Sweden there is a conservative approach to introducing new implants into routine use, and the revision rates for total hip and knee arthroplasties are very low. Several hip stems have shown very good long-term survival in national registries. When the use of hemiarthroplasties gained momentum in the early years of the past decade, the choice of implant was left to the discretion of the surgeon or the department since no registry data were available—and only limited data from clinical studies concerning modern hemiarthroplasties were available. Consequently, we saw a wide range of different implants in use during this period. With the increasing data on different implants, some of them are rapidly falling into disfavor. During the first years of recording, the Swedish Hip Arthroplasty Register identified a relatively high number of monoblock-type prostheses, mostly Austin-Moore and Thompson. Consistently poor results according to the SHAR (Garellick et al. 2010) in addition to those reported from the National Joint Replacement Registry in Australia (Graves et al. 2010) and from clinical trials (Jalovaara and Virkkunen 1991, Ravikumar and Marsh 2000, Blomfeldt et al. 2005) are probably the main reason for the disappearance of these designs from the Swedish market.

Contemporary uncemented stems are relatively common in Norway and Australia, and they account for 20% and 11% of the hemiarthroplasties implanted in these countries (Furnes and Gjertsen 2010, Graves et al. 2010). In the annual reports of the last years and at national orthopedics meetings, the SHAR has reported that there is an increased risk of re-operation with (contemporary) uncemented stems compared to cemented stems (Garellick et al. 2010). In accordance with these reports, the use of this type of stem has remained at a lower level in Sweden.

The most common reason for revision of a hemiarthroplasty is dislocation (Garellick et al. 2010). The use of an anterolateral approach reduces the risk of this complication (Chan and Hoskinson 1975, Montgomery and Lawson 1978, Enocson et al. 2008). In Sweden, there has been a trend towards use of this approach in THA for more than a decade (Garellick et al. 2010)—probably to address the risk of revision due to early dislocation—and in this study, we have seen the same trend for hemiarthroplasties.

The sudden decrease in the use of bipolar implant heads in 2009 is another interesting issue. In their annual report for 2008, the SHAR reported preliminary data showing that there was a higher risk of re-operation for bipolar heads (Garellick et al. 2009).There was also a suspicion that the problem was associated specifically with dislocations of the Vario Cup implant head. In the following year, a decreasing use of bipolar heads in general and of the Vario Cup in particular was noted.

The period 2005–2009 corresponds to the second phase of the introduction of hemiarthroplasty in Sweden. The idea of treating a displaced femoral neck fracture with hemiarthroplasty, as an alternative to internal fixation, won increasingly greater acceptance in the country from the late 1990s—with a swift increase until 2005, after which the increase has leveled off (Rogmark et al. 2010).

Regrettably, the records in the NPR lack laterality and it is impossible to establish the actual number of femoral neck fractures in Sweden. However, we can assume that the number of fractures per age group and sex did not change dramatically over the relatively short study period. We interpret the increase in hemiarthroplasty procedures—together with the increasing proportion of hemiarthroplasty patients with acute fracture and fewer salvage procedures after failed internal fixation—as being a reflection of the recent shift in treatment regime for femoral neck fractures.

The increased age in hemiarthroplasty patients reflects the fact that nowadays even individuals over 95 years old are treated with hemiarthroplasty (Rogmark et al. 2010). Overall, the population over 95 years of age in Sweden has increased from 11,000 in 1999 to 16,500 in 2009 (Statistics Sweden, SCB). With their very high risk of fracture, this growing elderly population group contributes to an increase in the absolute number of fractures (Bergstrom et al. 2009, Arakaki et al. 2011, Rosengren 2010). The predominant practice in Sweden for treatment of displaced femoral neck fractures is internal fixation or a total hip arthroplasty for the younger, medically fit patient and a hemiarthroplasty for the elderly, frailer patient. In addition, for very sick patients, surgeons are inclined to choose internal fixation as opposed to arthroplasty. Men with hip fractures generally have poorer health than women. The national quality register of hip fractures—Rikshöft—reports that 11% of the men with a hip fracture were classified as ASA 4 in 2009 as compared to 8% of the women (Thorngren 2010). At present, there is a trend to allow a higher degree of co-morbidity for arthroplasty eligibility, which might be a reason for the increasing male proportion (27–30%). The 30% figure for males in 2009 is in accordance with the Australian National Joint Replacement Registry, although the tendency in Australia is a somewhat decreasing proportion of males who undergo hemiarthroplasty (Graves et al. 2010).

As mentioned, local treatment guidelines often include extreme medical conditions as a reason for internal fixation instead of arthroplasty, given the reduced surgical trauma with the former method. This is reflected in our finding of fewer hemiarthroplasty patients with an ASA grade of 4 compared to the entire hip fracture group reported in the Rikshöft Annual Report (7% for men and 5% for women as compared to 11% and 8%, respectively) (Thorngren 2010).

Our finding of an increasing rate of cognitive impairment correlates well with the increasing age of hip fracture patients (Jorm and Jolley 1998). The Norwegian Hip Fracture Register uses the same simple classification of cognitive impairment. The mean age in the hip fracture group in Norway has been unchanged, and the rate of cognitive impairment has not increased during this period (Furnes and Gjertsen 2010). However, our conclusions might be limited by the incompleteness of the recordings of grade of cognitive impairment. Furthermore, we chose not to demand a validated score for this assessment—e.g. the Mini-Mental State examination (Folstein et al. 1975) or the Short Portable Mental State Questionnaire (Pfeiffer 1975)—as this might lead to an even lower degree of completeness of the recordings. Instead, the classification is done by the surgeon. There is a risk that patients with transient confusion could be falsely classified as having suspected or evident cognitive impairment, which also reduces the certainty of our conclusions.

The obvious weakness of large nationwide registrations is the lack of patient-reported results, such as subjective symptoms that do not lead to additional surgery. Patient-reported outcomes might be evaluated in smaller, preferably randomized studies. Another inherent weakness is the issue of selection bias. The choice of treatment method has been made by the treating surgeon, based on patient characteristics and the surgeon's preferences. Calculation of completeness is a special problem, as it is virtually impossible to ascertain the true number of procedures in the country with certainty. The major hindrance to this is the lack of laterality in the NPR. In addition, a number of patients might theoretically have undergone hemiathroplasty procedures without being registered in any of the registers (the SHAR or the NPR). However, reporting to the NPR is compulsory by law and the number of such cases can be assumed to be very low. Regarding re-operations, it is of course a disadvantage that closed reductions of dislocated hemiarthroplasties are not reported to the SHAR. The reason for this is previous experience (from the SHAR) of poor compliance in reporting of this complication.

Finally, the completeness concerning ASA grade has so far not been optimal. On the other hand, results from a nationwide prospective observational study can be generalized for the entire country and the whole of the Swedish orthopedic community. Such a large study can address unusual complications with satisfactory statistical power. Thus, national registries remain important tools in the continuing effort to improve patient care.

We conclude that the Swedish orthopedic surgeons continually modify their practice in order to ensure the best available treatment and care of patients. Findings and reports from the SHAR have most likely contributed to these changes.
